# Was verstehen Bürger*innen unter einem digitalen Setting im Kontext der Gesundheitsförderung? – Ergebnisse aus drei online-basierten World Cafés

**DOI:** 10.1007/s11553-023-01053-4

**Published:** 2023-06-08

**Authors:** Eleana Dongas, Anna Lea Stark, Joanna Albrecht, Kamil Joseph Wrona, Christoph Dockweiler

**Affiliations:** 1 Arbeitsgruppe Demografie und Gesundheit, Fakultät für Gesundheitswissenschaften, Universität Bielefeld, Universitätsstr. 25, 33615 Bielefeld, Deutschland; 2 Professur für Digital Public Health, Department Digitale Gesundheitswissenschaften und Biomedizin, Lebenswissenschaftliche Fakultät, Universität Siegen, Siegen, Deutschland; 3 Fachbereich Ingenieurwissenschaften und Mathematik, Hochschule Bielefeld, Bielefeld, Deutschland; 4 Fachbereich Gesundheit, Hochschule Bielefeld, Bielefeld, Deutschland

**Keywords:** Setting-Ansatz, Digitale Transformation, Bürgerverständnis, Definitionsableitung, Lebenswelt, Setting approach, Digital transformation, Citizens understanding, Definition deciration, Life world

## Abstract

**Hintergrund und Ziel:**

Durch die digitale Transformation haben digitale Maßnahmen zum Aufbau gesundheitsfördernder Strukturen in alltäglichen und beruflichen Settings an Bedeutung gewonnen. Zur Ableitung solcher Maßnahmen unter Berücksichtigung der Bedürfnisse von Setting-Mitgliedern soll eine Definition des Begriffs „digitales Setting“, inklusive der Aspekte des Gelingens bzw. Scheiterns der digitalen Transformation und Folgen für die Gesundheit und das Zusammenleben in Settings, aus Perspektive von Bürger*innen erarbeitet werden.

**Material und Methoden:**

Im August 2021 wurden drei online-basierte World Cafés, angelehnt an Brown & Isaacs, mit je maximal 13 Teilnehmenden aus den Settings Bildungseinrichtung, Kommune und Verein durchgeführt (*N*_gesamt_ = 34). Sie fanden online über Zoom und Conceptboard statt. Die Auswertung der Ergebnisse erfolgte mit MAXQDA, angelehnt an die strukturierende Inhaltsanalyse nach Kuckartz.

**Ergebnisse:**

Bürger*innen verwenden nicht den Begriff Setting, sondern Lebenswelt (LW). Unter einer digitalen LW verstehen sie ihre physisch vorhandene LW, in der in unterschiedlichem Ausmaß digitale Tools verwendet werden. Digitale LW bestehen Bürger*innen zufolge aus der digitalen Infrastruktur, Angeboten, Koordination/Planung, Informationsbereitstellung/-beschaffung und Kommunikation und sind immer als Kombination digitaler und analoger Bestandteile zu verstehen.

**Diskussion und Schlussfolgerung:**

Die hergeleitete Definition digitaler Settings aus Perspektive von Bürger*innen zeigt Anforderungen digitaler Interventionen, die in der Setting-bezogenen Gesundheitsförderung und Prävention künftig stärker berücksichtigt werden müssen. Durch deren Berücksichtigung im Leitfaden Prävention kann zu einer zielgruppen- und bedarfsorientierten Implementierung entsprechender Maßnahmen beigetragen werden.

## Abstract (en)

**Background and objectives:**

Due to digital transformation, digital interventions for developing health-promoting structures in everyday and professional settings have gained importance. In order to derive such interventions, considering the needs of setting members, a definition of the term *digital setting*, including aspects of the success or failure of the digital transformation and consequences on health and living together in settings, from the perspective of citizens will be developed.

**Materials and methods:**

In August 2021, three online World Cafés, based on Brown and Isaacs, were held with a maximum of 13 participants each from the settings of educational institution, community and leisure club (*N*_total_ = 34). They took place online via Zoom and Conceptboard. The results were analyzed using MAXQDA, based on structuring content analysis according to Kuckartz.

**Results:**

Citizens do not use the term setting, but living environment. By a digital living environment, they understand their physically existing living environment, in which digital tools are used to varying degrees. According to citizens, digital living environments consist of digital infrastructure, offers, coordination/planning, information provision/procurement and communication and are always to be understood as combining digital and analogue components.

**Discussion and conclusion:**

The derived definition of digital settings from a citizens’ perspective shows the requirements of digital interventions, which must be taken more into account in setting-related health promotion and prevention in the future. Taking them into account in the “Leitfaden Prävention” (prevention guideline) can contribute to a target-group- and needs-oriented implementation of corresponding interventions.

## Hintergrund

Angesichts des demografischen Wandels, des medizinisch-technischen Fortschritts und der fortschreitenden digitalen Transformation (DT) haben sich die Anforderungen an eine effektive Gesundheitsförderung (GF) und Prävention gewandelt [20]. Digitale Gesundheitstechnologien wirken sich nachweislich positiv auf die Gesundheit aus [6, 8, 15, 16]. Aufgrund ihrer hohen Reichweite, Effektivität, Niederschwelligkeit und der Zeit- und Kostenersparnis bieten sie ein großes Potenzial [12, 20, 21] und eignen sich zur Setting-bezogenen GF [5].

Um dieses Potenzial bestmöglich für die Setting-bezogene GF nutzen zu können, muss vorerst untersucht werden, wie sich Settings digital transformieren und was dies für die Setting-bezogene GF und Prävention bedeutet. Settings sind entlang der Definition der Weltgesundheitsorganisation „the place or social context in which people engage in daily activities in which environmental, organizational, and personal factors interact to affect health and wellbeing“ [7].^1^ Sich neu bildende bzw. zunehmend digitale Settings, wie z. B. eSport-Vereine, könnten gesundheitsfördernde (z. B. Fitnesssteigerung), aber auch pathogene Effekte (z. B. Cyberbullying) haben [11, 12, 19]. Die Ableitung von Interventionen zum Aufbau gesundheitsfördernder Strukturen in digitalen Settings ist daher von hoher Relevanz. Voraussetzung ist die Entwicklung einer politisch und praktisch operationalisierbaren Definition des Begriffs *digitales Setting*, die die Bedürfnisse von Setting-Mitgliedern berücksichtigt.

Die Studie zielt darauf ab, eine Definition des Begriffs *digitales Setting* im Kontext von Prävention und GF aus Perspektive von Bürger*innen zu erarbeiten. Bürger*innen sind Mitglieder von Settings und formen diese permanent, weshalb ihr Verständnis von digitalen Settings besonders relevant ist. Es soll untersucht werden, welche Aspekte Bürger*innen zufolge zum Gelingen bzw. Scheitern der DT in Settings beitragen und welche Folgen diese auf die Gesundheit und das Zusammenleben in Settings hat. Somit könnten digitale gesundheitsfördernde Interventionen künftig an die Bedürfnisse von Bürger*innen angepasst und möglichst effektiv gestaltet werden. Die handlungsleitende Fragestellung lautet vor diesem Hintergrund: „Wie kann der Begriff *digitales Setting* im Kontext der GF und Prävention aus Perspektive von Bürger*innen definiert werden?“

## Material und Methoden

Im August 2021 wurden drei online-basierte World Cafés mit je maximal 13 Teilnehmenden aus den Settings Bildungseinrichtung, Kommune und Verein durchgeführt. Diese sind im Leitfaden Prävention verankert und ermöglichen durch ihre Heterogenität die Generierung eines umfassenden Verständnisses von digitalen Settings. Die Teilnehmenden stammen aus verschiedenen Einrichtungen (s. Stichprobenbeschreibung) und Statusgruppen (s. Tab. 1), um auch innerhalb der Settings möglichst vielschichtige Teilstichproben zu erhalten. Aufgrund vermehrter Absagen konnten Jugendfreizeiteinrichtungen nicht, wie ursprünglich geplant, in das Setting *Verein* eingeschlossen werden, sodass dieses nur Sport- und Senior*innenvereine umfasste und somit weniger heterogen war als die Settings *Bildungseinrichtung* und *Kommune*. Die Teilnehmenden wurden per E‑Mail, z. T. über Leitungspersonen aus Einrichtungen, rekrutiert. Zur Gewährleistung der Perspektivendiversität wurden die Gruppen hinsichtlich des Geschlechts, Alters und beruflichen Status durchmischt. Die World Cafés dauerten je ca. 3 h (Durchschnittsdauer = 191 min).

**Tab. 1:** Soziodemografische Angaben des Samples (*n* = 34)

Variable	Ausprägungen	Häufigkeiten (%)
Altersgruppe	20–29	47,1
	30–39	14,7
	40–49	14,7
	50–59	11,8
	60–69	8,8
	70–79	2,9
Geschlecht	Männlich	38,2
	Weiblich	61,8
	Divers	0,0
Statusgruppe	(Bereichs‑)Leitung/Koordinator*in	24,3
	Angebots‑/Kursleitung	18,9
	Verwaltungstechnische Mitarbeitende	8,1
	Angebots‑/Kursteilnehmende	48,6

### Methode und Online-Umsetzung der World Cafés

Die Online-World Cafés wurden in Anlehnung an Brown und Isaacs [2] durchgeführt. Dabei führt eine größere Gruppe an Teilnehmenden an kleineren, wechselnden Tischgruppen aufeinander aufbauende Gespräche. So werden Ideen weitergetragen, Perspektiven verknüpft und kollektives Wissen generiert [2].

Die World Cafés fanden online über *Zoom* (Zoom Video Communications Inc., San José, CA, USA) und die digitale Pinnwand *Conceptboard* (Conceptboard Cloud Service GmbH, Halle, Deutschland) statt, auf der Teilnehmende ihre Diskussionspunkte visuell festhalten konnten. Sie bearbeiteten Fragen im Plenum und in Kleingruppen (via Breakout-Räume in Zoom) hinsichtlich der Elemente eines digitalen Settings, Aspekten des Gelingens/Scheiterns der DT und Folgen für die Gesundheit und das Zusammenleben in Settings. Die entsprechenden Fragen^2^ lauteten:

Tischfragen:

1. Inwiefern spielt die Digitalisierung eine Rolle für das alltägliche Leben in unserer Stadt?
2. Was würde eine digitale Stadt ausmachen?
3. Was trägt zum Gelingen oder Scheitern der Digitalisierung in einer Stadt bei?
4. Wie wirkt sich die Digitalisierung in einer Stadt auf das alltägliche Leben und die Gesundheit aus?

Plenumsfrage:

5. Was verstehen wir unter einer digitalen Stadt?

Die Fragen wurden in einem Pre-Test auf Verständlichkeit geprüft und angepasst. Die Teilnehmenden wurden im Vorhinein hinsichtlich des Datenschutzes und der Freiwilligkeit der Teilnahme aufgeklärt. Nach dem ersten World Café wurde das methodische Vorgehen modifiziert (nur zwei Kleingruppenwechsel, Kürzung der Arbeitsphase im Plenum, Fragenformulierungen). Über einen Kurzfragebogen wurden soziodemografische Angaben und die Technikaffinität [9] erhoben. Zusätzlich zu den Notizen auf dem *Conceptboard* fertigten die Forscher*innen Gedächtnisprotokolle an [22].^3^

### Datenanalyse und -auswertung

Zur Beschreibung der Stichprobe wurde der Kurzfragebogen mittels deskriptiver Häufigkeitsanalysen ausgewertet. Die *Conceptboards* und Gedächtnisprotokolle wurden mit *MAXQDA* (VERBI – Software. Consult. Sozialforschung. GmbH, Berlin, Deutschland), angelehnt an die strukturierende Inhaltsanalyse nach Kuckartz [10], ausgewertet. Auf dieser Basis wurden Setting-spezifische Gemeinsamkeiten und Unterschiede ermittelt und zur übergreifenden Definitionsentwicklung herangezogen.

## Ergebnisse

### Beschreibung der Stichprobe

An der World Café *Bildungseinrichtung* nahmen 13 Personen aus Einrichtungen der Erwachsenenbildung und (Hoch‑)Schulen teil, am World Café *Kommune *12 Teilnehmende aus dem Quartiersmanagement, der kommunalen Verwaltung und der Bürgerschaft und am World Café *Verein* 9 Personen aus Sport- und Senior*innenvereinen. 47,1 % der Teilnehmenden sind zwischen 20 und 29 Jahre alt (s. Tab. 1) und 61,8 % weiblich. Folgende Statusgruppen liegen vor: (Bereichs‑)Leitung/Koordinator*in (24,3 %), Angebots‑/Kursleitung (18,9 %), verwaltungstechnische Mitarbeitende (8,1 %) und Angebots‑/Kursteilnehmende (48,6 %).^4^

### Begriffsverständnis einer digitalen Lebenswelt

Unter einer digitalen Lebenswelt (LW) verstehen Bürger*innen ihre analoge bzw. physisch vorhandene LW, in der in unterschiedlichem Ausmaß digitale Tools verwendet werden. Bürger*innen verwenden nicht den Begriff *Setting*, sondern *Lebenswelt* oder benennen die LW bei ihrem konkreten Namen (z. B. Verein). Sie verstehen eine digitale LW immer als Kombination digitaler und analoger Bestandteile.

#### Strukturmerkmale einer digitalen LW

Aus Perspektive von Bürger*innen setzt sich eine digitale LW aus der digitalen Infrastruktur (digitale Endgeräte, WLAN, Strom etc.) als Basis und vier Strukturmerkmalen zusammen. Dazu zählen teilweise digitalisierte Angebote, die in LW verfügbar sind, z. B. digitalisierte Bildungs- und kommunale Angebote (eShopping, eSport, eBehörden, digitale kulturelle/gesundheitliche Angebote etc.). Hinzu kommen die digitalisierte Koordination und Planung, bestehend aus digitalen Tools zur alltäglichen und institutionellen Organisation von Aufgaben oder Optimierung von Prozessen in LW (z. B. Online-Terminkalender, digitalisierte Kursplanung etc.). Durch diese Tools wird die Kommunikation vereinfacht, Prozesse optimiert und eine einfachere Koordination von Veranstaltungen ermöglicht.

Weiter umfasse eine digitale LW die digitalisierte Informationsbereitstellung und -beschaffung. Es sei ein „offener Datenraum“ entstanden, über den Informationen in LW jederzeit und überall angeboten, abgerufen und miteinander verglichen werden können. Bürger*innen erachten auf die User*innen abgestimmte Apps, Websites und Plattformen und die schnellere Informationsvermittlung/‑verbreitung als hilfreiche Bestandteile ihrer digitalen LW. Zudem zeichne sich eine digitale LW durch Kommunikation und sozialen Austausch über digitale Tools aus. Online-Foren, virtuelle Meetingräume, Videokonferenzsysteme und Chat-Tools ermöglichen demnach eine ortsunabhängige sowie zeitlich flexiblere Kommunikation und fördern den sozialen Austausch.

#### Hybridität

Bürger*innen verstehen digitale Tools als Ergänzung, nicht als Ersatz ihrer analogen LW und bezeichnen sie entsprechend als hybrid. Der Einsatz digitaler Tools sei nicht immer in allen Bereichen gewünscht bzw. möglich. Er weise z. T. Grenzen bzw. Nachteile auf (z. B. den Verlust des Zwischenmenschlichen). Bürger*innen betonen die Relevanz einer Balance zwischen Analog und Digital: „Präsenz und Digital nicht gegeneinander, sondern miteinander“ (Bürgerin, World Café *Bildungseinrichtung*). Die SARS-CoV-2-Pandemie („severe acute respiratory syndrome coronavirus 2“) habe die Entwicklung hin zu Hybridlösungen enorm vorangetrieben.

### Aspekte des Gelingens bzw. Scheiterns der digitalen Transformation in LW

**Abb. 1:**
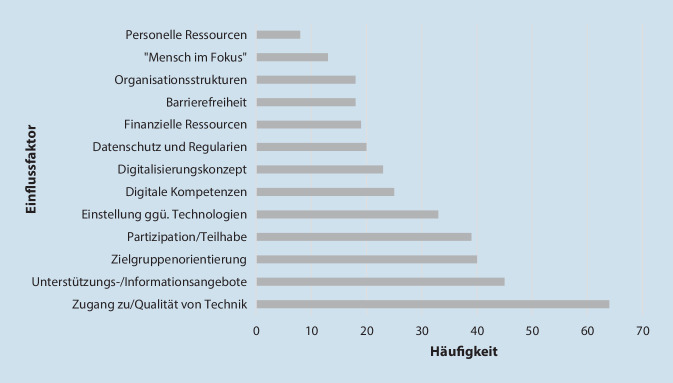
Priorisierung der Aspekte des Gelingens bzw. Scheiterns der digitalen Transformation in Lebenswelten nach Anzahl der Nennung und Verschriftlichung

Bürger*innen benennen verschiedene Aspekte, die zum Gelingen bzw. Scheitern der DT in LW führen können (s. Abb. 1). Sie beziehen sich sowohl auf die Verhaltens- als auch Verhältnisebene ihrer LW.

Als besonders relevant für eine gelingende DT in LW erachten Bürger*innen den Zugang zu bzw. die Qualität der genutzten Technik und entsprechende Unterstützungs‑/Informationsangebote (z. B. wohnortnahe Beratung zu Internet-Gefahren). Besonders Peer-to-peer-Angebote (z. B. Digitalisierungspaten) und die intergenerationelle Unterstützung stellen großes Potenzial dar. Solche Angebote könnten Beteiligte zur Nutzung digitaler Tools befähigen, den Umgang mit diesen erleichtern und Ängste/Vorbehalte abbauen. Basis für eine gelingende DT stellen zudem die technischen Voraussetzungen (WLAN, Stromversorgung, Hard‑/Software) dar. Sie sollten eine hohe Qualität aufweisen und barrierearm gestaltet sein.

Angesichts z. T. bestehender alters-/persönlichkeitsspezifischer Unterschiede zwischen LW-Mitgliedern sollte die DT zielgruppenorientiert gestaltet sein und den „Mensch(en) im Fokus“ haben (Bürger, World Café *Bildungseinrichtung*). Es brauche bei der Konzeption, Umsetzung und Evaluation von Angeboten eine Differenzierung nach Zielgruppen und ihren Bedürfnissen und Kompetenzen sowie die Partizipation aller Mitglieder. Man sollte die „Zielgruppe von Anfang an bei Planung und Konzeption von Angeboten im Blick haben“ (Bürgerin, World Café *Bildungseinrichtung*). So könne die DT ausgebaut, Chancengleichheit gefördert und Ausgrenzung vorgebeugt werden.

Aus Perspektive der Bürger*innen spielen eine positive Einstellung gegenüber Technologien und ausreichende digitale Kompetenzen aller LW-Mitglieder eine Rolle für eine gelingende DT in LW. Auf verhältnisbezogener Ebene seien zudem ein transparentes Digitalisierungskonzept, ausreichender Datenschutz und Regularien sowie förderliche Organisationsstrukturen (insbesondere Agilität) von Relevanz. Organisationen sollten im Digitalisierungsprozess lernen sowie flexibel und proaktiv agieren. Auch die Barrierefreiheit von Technologien und finanzielle/personelle Ressourcen (z. B. Digitalisierungspaten) für die Ausgestaltung der DT und Implementierung entsprechender Angebote in LW trügen zum Gelingen der DT bei.

### Potenziale und Risiken der digitalen Transformation von LW

**Abb. 2:**
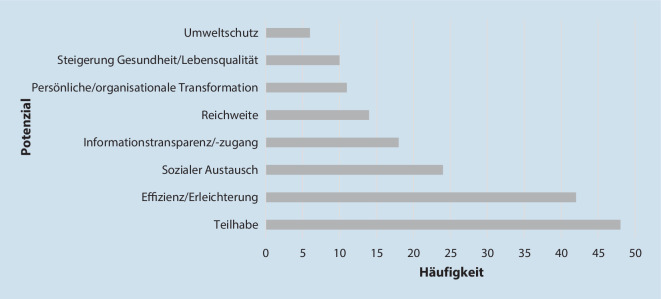
Priorisierung der Potenziale der digitalen Transformation von Lebenswelten nach Anzahl der Nennung und Verschriftlichung

Mit dem Einsatz digitaler Tools verbinden Bürger*innen sowohl Potenziale als auch Risiken hinsichtlich der Gesundheit und des Zusammenlebens in LW (s. Abb. 2 und 3). Als vorteilhaft erachten sie verbesserte Möglichkeiten der Teilhabe und des sozialen Austauschs. Durch den Einsatz von Technologien und die damit einhergehenden niedrigschwelligeren Optionen der Teilhabe an Angeboten in LW könnten Barrieren abgebaut werden. Beispielsweise bestehe eine „niedrigere Hemmschwelle, sich für einen Online-Kurs anzumelden“ (Bürger, World Café *Verein*). Durch vereinfachte digitale Kommunikationsmöglichkeiten könne zudem Gemeinschaft gefördert, ein gestärkter Zusammenhalt erzeugt, LW-Mitglieder zur (regelmäßigen) Teilnahme an digitalen Angeboten motiviert und das Zusammensein belebt werden.

**Abb. 3:**
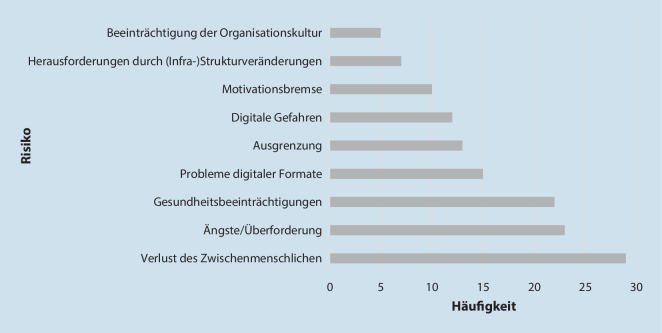
Priorisierung der Risiken der digitalen Transformation von Lebenswelten nach Anzahl der Nennung und Verschriftlichung

Des Weiteren erleben Bürger*innen die Erleichterung im Alltag und das effizientere Ausführen von Aufgaben (z. B. Terminplanung) als Potenzial der DT in LW. Auch die höhere Informationstransparenz bzw. den besseren Informationszugang, die größere Reichweite digitaler Angebote und die Optionen der persönlichen/organisationalen Transformation nehmen sie als positiv wahr. Auf individueller Ebene könne durch das Wahrnehmen von digitalen Angeboten bspw. eine Stärkung des Selbstbewusstseins und der Unabhängigkeit entstehen. Auf organisationaler Ebene könnten sich neue digitale Angebote (z. B. neue eSportarten) bilden und Organisationsstrukturen und -kultur positiv verändern (z. B. durch Prozessoptimierung).

Die Steigerung der Gesundheit bzw. Lebensqualität und der Umweltschutz stellen weitere Potenziale der DT in LW dar. Durch die ortsunabhängige Teilnahme an digitalen Angeboten könnten unnötige Fahrtwege vermieden, die Umwelt geschützt, Stress reduziert und die mentale Gesundheit gefördert werden. Die Erleichterung im Alltag, z. B. die „Stressreduzierung durch digitale Sitzungen“ (Bürgerin, World Café *Verein*), und die Optionen des digitalen sozialen Austauschs könnten sich auch positiv auf die Lebensqualität auswirken.

Risiken der DT von LW sehen Bürger*innen insb. in dem Verlust des Zwischenmenschlichen, Ängsten/Überforderung (z. B. durch mangelnden Datenschutz) und physischen/psychischen Gesundheitsbeeinträchtigungen (z. B. Bewegungsmangel, mentale Belastung). So werde das soziale Miteinander bei der Nutzung digitaler Tools eingeschränkt: „Persönlicher Kontakt bietet mehr als digital(er)“ (Bürgerin, World Café *Kommune*). Zudem bestünde die Gefahr des sozialen Ausschlusses und Vereinsamung, was sich negativ auf die Gesundheit auswirken könne. In diesem Kontext bestünde ein Zusammenhang zwischen ungleichen digitalen Voraussetzungen und gesundheitlicher Ungleichheit. Daher sei es nötig, Chancengleichheit im Rahmen der DT von LW zu schaffen, digitale Kompetenzen zu fördern und auch analoge soziale Bindungen zu erhalten.

Auf Verhältnisebene nehmen Bürger*innen Probleme digitaler Formate (z. B. Verlust des Zwischenmenschlichen) bzw. digitale Gefahren (z. B. Cyberbullying), Herausforderungen durch Strukturveränderungen (z. B. Verlust regionaler Geschäfte) und Beeinträchtigungen der Organisationskultur (z. B. Probleme bei Aufbau/Erhalt der Vereinskultur) als Risiken der DT wahr. Auch könne es in digitalen LW zu einem Motivationsverlust zur Teilnahme an digitalen Angeboten kommen: „online kann eine Motivationsbremse sein“ (Bürger, World Café *Verein*).

## Diskussion

In Bezug auf die Forschungsfrage kann gefolgert werden, dass Bürger*innen nicht den Begriff *Setting*, sondern *Lebenswelt* verwenden. Sie verstehen eine digitale LW als Kombination digitaler und analoger Bestandteile bzw. als hybrid. Eine rein digitale LW halten sie für wenig wünschenswert und unrealistisch.

Bürger*innen nennen diverse Aspekte, die zum Gelingen der DT ihrer LW beitragen. Besonders die benannten Aspekte auf Verhältnisebene, das Vorhandensein eines Digitalisierungskonzepts, förderlicher Organisationsstrukturen (wie Agilität), ausreichend finanzieller Ressourcen und ein umfassender Datenschutz werden auch in anderen Studien als wichtige Aspekte des Gelingens der DT in Organisationen beschrieben [3, 4, 13]. Weiter nennen Bürger*innen Potenziale, die sie der DT in LW zuschreiben, z. B. die bessere Teilhabe an Angeboten, die Option des sozialen Austauschs und die Effizienz bei der Ausführung von Aufgaben. Als Risiken erachten sie den Verlust des Zwischenmenschlichen und potenziell auftretende Gesundheitsbeeinträchtigungen. Auch dies steht im Einklang mit den Erkenntnissen bisheriger Studien, die insbesondere psychische Folgen wie Depressionen oder Suchtverhalten, den im Zuge des beeinträchtigten sozialen Austauschs auftretenden Verlust von *Soft Skills* und die von Bürger*innen benannte Gefahr der Ausgrenzung (*Digital Divide) *als Risiken der DT beschreiben [11, 14, 17].

Die hergeleitete Definition *digitaler Settings *aus Perspektive von Bürger*innen zeigt die Anpassungsbedarfe gesundheitsfördernder Maßnahmen in einer sich immer stärker digitalisierenden Welt, die in der Setting-bezogenen GF und Prävention künftig stärker berücksichtigt werden müssen. Sie ermöglicht eine zielgruppen- und bedarfsorientierte Implementierung entsprechender Interventionen und sollte demnach im Leitfaden Prävention sowie weiteren gesetzlichen Grundlagen der Ausgestaltung von gesundheitsfördernden Maßnahmen berücksichtigt werden. Dies ist besonders im Kontext der Organisationsentwicklung von Bedeutung, da die DT hier mit z. T. tiefgreifenden Veränderungen der Prozesse und Strukturen einhergeht und somit auch Veränderung im Alltags- und Arbeitsleben nach sich zieht, was mit Belastungen für Setting-Mitglieder einhergehen kann [18]. Die von Bürger*innen wahrgenommenen Strukturmerkmale und Potenziale bzw. Risiken ihrer digitalen LW sind daher für die Anpassung künftiger Maßnahmen der Setting-bezogenen GF und Prävention von großer Relevanz.

Es fällt auf, dass in der Wahrnehmung der Potenziale und Risiken z. T. Divergenzen bei Bürger*innen erkennbar sind. So wird z. B. die DT einerseits als gesundheitsfördernd wahrgenommen, andererseits als gesundheitsbeeinträchtigend. Derartige Differenzen lassen sich vermutlich auf persönliche Faktoren zurückführen und sind nicht einheitlich auf eine Gesamtgruppe übertragbar, was die Relevanz der Zielgruppenspezifität bei der Ausgestaltung der DT in Settings unterstreicht und Raum für weitere Forschung bietet.

## Limitationen

Die Ergebnisse müssen hinsichtlich limitierender Faktoren interpretiert werden. Zum einen konnten *Jugendfreizeitvereine* aufgrund vermehrter Absagen nicht eingeschlossen werden, wodurch das Setting *Verein* weniger heterogen war. Die mit dem Online-Format einhergehenden Anforderungen können zudem zu einem Rekrutierungsbias technikaffinerer Teilnehmender geführt haben und weitere tendenziell vulnerable Personengruppen ausgeschlossen haben. Zum anderen haben zwischenzeitige technische Störungen während der Online-World Cafés den Diskussionsfluss z. T. beeinträchtigt. Für derartige Probleme konnte jedoch schnell eine Lösung gefunden werden, sodass sie die Ergebnisse vorrausichtlich nicht beeinflusst haben^5^.

## Fazit für die Praxis

- Die Ergebnisse zeigen, was Bürger*innen im Kontext der GF unter einem *digitalen Setting* verstehen. Hieraus lassen sich folgende Handlungsempfehlungen ableiten:
- Es sollten geeignete Einsatzbereiche (z. B. digitalisierte Sportvereine etc.) und Technologien identifiziert und nötige Rahmenbedingungen sowie (gesundheitsbezogene) Chancen und Risiken der digitalen Transformation (DT) bei der Ableitung gesundheitsfördernder Maßnahmen in Settings berücksichtigt werden.
- Die Fokussierung des Menschen inklusive seiner Bedürfnisse sollte bei der Ausgestaltung der DT und der Ableitung gesundheitsfördernder (digitaler) Interventionen in Settings sichergestellt werden.
- Neben niedrigschwelligen Zugängen zu einer digitalen Infrastruktur sollte zielgruppen- und bedarfsorientierte Unterstützung im Setting angeboten werden.
- Um Exklusion zu vermeiden und Bedürfnissen gerecht zu werden, sollten hybride Formate bei der Ausgestaltung gesundheitsfördernder Maßnahmen in Settings angeboten werden und digitale Kompetenzen berücksichtigt bzw. gefördert werden.

## Footnotes

1 Synonym zu diesem Begriff wird im Präventionsgesetz der Begriff Lebenswelt verwendet [5]. Auch die Teilnehmenden der vorliegenden Studie nutzen zur Bezeichnung eines Settings den Begriff Lebenswelt. Zur authentischen Wiedergabe der Teilnehmendenperspektive wird dieser Begriff daher für die Ergebnisdarstellung in diesem Artikel übernommen. Ansonsten wird der Begriff Setting verwendet.

2 Die Fragestellungen sind hier beispielhaft für das Setting *Kommune* dargestellt und wurden an die anderen Settings entsprechend angepasst.

3 Eine ausführliche Beschreibung des methodischen Vorgehens ist einem separaten Artikel zu entnehmen [1].

4 Z. T. gehören Teilnehmende mehreren Statusgruppen gleichzeitig an.

5 Eine ausführliche Beschreibung der methodischen Limitationen ist einem separaten Artikel zu entnehmen [1].

## References

[1] Albrecht J, Stark AL, Dongas E, Wrona KJ, Dockweiler C (2022) Hosting an online world Café to develop an understanding of digital health promoting settings from a citizen’s perspective—methodological potentials and challenges. Int J Environ Res Public Health 19(16):9969. 10.3390/ijerph19169969 36011601 10.3390/ijerph19169969 PMC9408369

[2] Brown J, Issacs D (2005) World café: shaping our futures through conversations that matter. Berrett-Koehler, San Francisco

[3] Cichosz M, Wallenburg CM, Knemeyer AM (2020) Digital transformation at logistics service providers: barriers, success factors and leading practices. Int J Logist Manag 31(2):209–238. 10.1108/IJLM-08-2019-0229

[4] https://scholarspace.manoa.hawaii.edu/server/api/core/bitstreams/cef25334-2ba8-48df-ab18-b274ccd4e747/content. Zugegriffen: 1. Juli 2022

[5] https://www.sozialgesetzbuch-sgb.de/sgbv/20a.html. Zugegriffen: 15. Juli 2022

[6] https://www.who.int/reproductivehealth/publications/digital-interventions-health-system-strengthening/en/. Zugegriffen: 1. Juli 2022

[7] https://www.who.int/teams/health-promotion/enhanced-wellbeing/healthy-settings. Zugegriffen: 15. Juli 2022

[8] Jacobs RJ, Lou JQ, Ownby RL, Caballero J (2016) A systematic review of eHealth interventions to improve health literacy. Health Informatics J 22(2):81–98. 10.1177/1460458214534092 24916567 10.1177/1460458214534092

[9] Karrer-Gauß K, Glaser C, Clemens C, Bruder C (2009) Technikaffinität erfassen – der Fragebogen TA-EG. ZMMS Spektrum 29:194–199

[10] Kuckartz U (2018) Qualitative Inhaltsanalyse. Methoden, Praxis, Computerunterstützung, 4. Aufl. Beltz, Weinheim

[11] Kuznetsova V, Azhmukhamedov I (2020) Advantages and risks of using the digital educational environment. ARPHA Proc 3:1369–1381. 10.3897/ap.2.e1369

[12] Lupton D (2014) Health promotion in the digital era: a critical commentary. Health Promot Int 30(1):174–183. 10.1093/heapro/dau091 25320120 10.1093/heapro/dau091

[13] Mhlungu NSM, Chen JYJ, Alkema P (2019) The underlying factors of a successful organisational digital transformation. S Afr J Inf Manag 21(1):a995. 10.4102/sajim.v21i1.995

[14] Müller AC, Wachtler B, Lampert T (2020) Digital Divide – Soziale Unterschiede in der Nutzung digitaler Gesundheitsangebote. Bundesgesundheitsblatt Gesundheitsforschung Gesundheitsschutz 63:185–191. 10.1007/s00103-019-03081-y 31915863 10.1007/s00103-019-03081-y PMC8057990

[15] Nour M, Chen J, Allman-Farinelli M (2016) Efficacy and external validity of electronic and mobile phone-based interventions promoting vegetable intake in young adults: systematic review and meta-analysis. J Med Internet Res 18(4):e58. 10.2196/jmir.5082 27059765 10.2196/jmir.5082 PMC4841894

[16] Pakarinen A, Parisod H, Smed J, Salanterä S (2017) Health game interventions to enhance physical activity self-efficacy of children: a quantitative systematic review. J Adv Nurs 73(4):794–811. 10.1111/jan.13160 27688056 10.1111/jan.13160

[17] Parisod H, Aromaa M, Kauhanen L, Kimppa K, Laaksonen C, Leppänen V, Pakarinen A, Smed J, Salanterä S (2014) The advantages and limitations of digital games in children’s health promotion. Finnish J eHealth eWelfare 6(4):164–173 (https://journal.fi/finjehew/article/view/48210)

[18] Pieck N, Held U, Bindl C (2019) Digitalisierung aus der Perspektive der gesundheitsfördernden Organisationsentwicklung. In: Badura B, Ducki A, Schröder H, Klose J, Meyer M (Hrsg) Fehlzeiten-Report 2019: Digitalisierung – gesundes Arbeiten ermöglichen, 1. Aufl. Springer, Berlin Heidelberg

[19] Quennerstedt M (2019) Social media as a health resource. In: Goodyear VA, Armour K (Hrsg) Young people, social media and health. Routledge, London, New York, S 71–85

[20] Stellefson M, Paige SR, Chaney BH, Chaney JD (2020) Evolving role of social media in health promotion: updated responsibilities for health education specialists. Int J Environ Res Public Health 17(4):1153. 10.3390/ijerph17041153 32059561 10.3390/ijerph17041153 PMC7068576

[21] Taggart T, Grewe ME, Conserve DF, Gliwa C, Roman Isler M (2015) Social media and HIV: a systematic review of uses of social media in HIV communication. J Med Internet Res 17(11):e248. 10.2196/jmir.4387 26525289 10.2196/jmir.4387 PMC4642795

[22] Vogel D, Funck BJ (2018) Immer nur die zweitbeste Lösung? Protokolle als Dokumentationsmethode für qualitative Interviews. Forum Qual Sozialforsch 19(1):1–29. 10.17169/fqs-19.1.2716

